# Shisha use among students in a private university in Kigali city, Rwanda: prevalence and associated factors

**DOI:** 10.1186/s12889-018-5596-1

**Published:** 2018-06-08

**Authors:** Omoboriowo Joad Omotehinwa, Ogendi Japheths, Iyamuremye Jean Damascene, Michael Habtu

**Affiliations:** 1 Department of Public Health, Mount Kenya University, School of Health Sciences, P. O. Box 5826, Kigali Campus, Kigali, Rwanda; 2 Rwanda Biomedical Center, Ministry of Health Kigali, Kigali, Rwanda

**Keywords:** Knowledge, Attitude, Shisha smoking, Prevalence, University students, Kigali city, Rwanda

## Abstract

**Background:**

All over the globe shisha smoking is fast growing among different age brackets. Shisha use has been reported to be increasing among youths in African major cities. Its use is documented to result in health effects such as lung cancer, cardiovascular and respiratory conditions, periodontal diseases, keratoacanthoma which are also associated with cigarette smoking. In Kigali, Rwanda’s capital, reports indicate that shisha use is increasing among the youths, particularly the university students. The study aimed at determining the prevalence, and establishing factors associated with shisha use among students in a university in Kigali Rwanda, which will be a significant step in stemming shisha fame among youths in Rwanda as there was no previously documented evidence-based study.

**Methods:**

A total of 427 students were selected for this cross-sectional study using stratified sampling method. A questionnaire was used to collect data on shisha use, knowledge about shisha, attitude towards shisha, and factors associated with use of shisha. The association between the independent variables and shisha use was assessed using chi-square *p* value < 0.05. Binary logistic regression was used to determine variables that were independently associated with shisha smoking.

**Results:**

Prevalence of ever smoking shisha among the university students was 26.1% and that of those that smoked in the last month (30 days) was 20.8%. About 40 % (39.9%) of the participants that had heard about shisha before demonstrated low level of knowledge, and 41.6% of these reported shisha use in the last 30 days. Logistic regression revealed that the followings were independently associated with shisha smoking: always drink alcohol (*p* = 0.003); drink alcohol occasionally (*p* = 0.045); having friend(s) who smoke shisha (*p* = 0.001); being aware of shisha’s availability in cafes, bars and restaurants in Kigali (*p* = 0.022); positive attitude towards shisha smoking (*p* < 0.001) and participants with age < 20 years (*p* = 0.039).

**Conclusions:**

There is a relative high prevalence of shisha smoking and a poor knowledge about its impact on health among these university students. Regular medical education and health promotion targeting the youths could improve knowledge and practices about shisha use. Shisha laws and regulations should be enacted, and fines imposed on individuals or group who flout them.

**Electronic supplementary material:**

The online version of this article (10.1186/s12889-018-5596-1) contains supplementary material, which is available to authorized users.

## Background

Tobacco use causes nearly six million deaths globally [1]. It is projected that the attributable mortality due to tobacco smoking will increase from 3 million deaths in 1990 to 8.4 million deaths in 2020 [2]. Tobacco smoking is estimated to cost over US $500 billion in economic damage each year globally [3].

Shisha, a form of tobacco smoking, has emerged as a global public health concern and has been described as the ‘emerging deadly trend’ [4]. Shisha smoking has been significantly linked with similar diseases attributable to cigarette smoking [5] and even more [6]. The aerosol of shisha smoke is reported to have high concentrations of carbon monoxide, nicotine, tar, and heavy metals at concentrations which are as high as or higher than those among cigarette smokers and which are toxic to the human body [7–9]. The practice of shisha smoking is also reported to be capable of spreading infections such as tuberculosis, mononucleosis, viruses and bacteria when the mouth piece is shared [10–12].

It has been documented that shisha smoking is common among the youth mainly high school children, college and university students [13]. Over 60% of students reported that they had used or were using shisha at the time of a study in a disadvantage secondary school in Johannesburg [14]. Sixty-six percent (66%) had smoked shisha before and 18% were current smokers among health science students of the University of Cape Town [15]. High prevalence (36.4%) of shisha smoking and low knowledge regarding its health effects was reported in youths attending bars in Kampala Uganda [16]. Amongst Sudanese tuberculosis patients and Fayoum University students in Egypt, 7.9 and 8.3% participants were shisha smokers respectively [17, 18]. The prevalence of shisha smoking among a United States college population was 64% for ever smoked shisha and 34% for smoked shisha within the previous 30 days [19]. Similarly, a study among university students in Beirut revealed that ever shisha smokers were 43% and current shisha smokers were 28.3% [20].

In Rwanda, shisha smoking has been described as the “the new craze in Kigali” [21] with the local dailies also describing it as a way of death [22].

Despite these reported concerns about the increasing popularity of shisha smoking amongst the youth in Kigali City of Rwanda, true prevalence of shisha smoking and factors associated with shisha use among the youths, remains unknown. In Rwanda, the government document on control of tobacco is silent on shisha control [23]. Studies on students in Rwanda have addressed Human Immunodeficiency Virus [24, 25] and reproductive health issues [26]. No study on Shisha smoking has focused on this sub population. The National school health policy in Rwanda [27] which outlines notable health problems among students and cited needed approach to address them was also silent on shisha smoking. In December 2017, the Government of Rwanda abolished smoking of shisha in Rwanda and importation of the products for use in shisha smoking [28], in line with World Health Organization Guidelines on Tobacco Use [29]. However, the current prevalence which would act as a baseline data to assess the effect of the ban on shisha smoking in this sub group is lacking.

The purpose of this study was to determine the prevalence and factors associated with shisha use among university students in the city of Kigali. Understanding the prevalence of shisha smoking, knowledge of its health effects and factors associated with shisha use will be of use to practitioners in designing the intervention strategies to this vulnerable group of the population.

## Methods

### Design and study population

This was a cross sectional study in which information on the prevalence of shisha use, knowledge of shisha effects, and factors associated with the use of shisha was sought among students from Mount Kenya University, Kigali city Rwanda. Kigali, the capital city of Rwanda, has a population of 1.13 million. Rwanda is a low income country with a gross national income per capita of $710.0 [30]. It is one of the countries that form the East African community. The University had a population of 2764 students during the 2016/2017 academic year [31]. The University has four faculties namely: School of Business and social sciences; School of Pure and Applied sciences; School of Health sciences, and School of Education. The University’s activities are during the day, in the evenings and on weekends to suit the demand of its prospective students [31].

### Sample size and sampling technique

A total of 427 respondents was calculated using the Cochran formula [32], *n* = Z ^2^pq / e^2^ whereby: n is the sample size; Z is the value for the selected alpha level 1.96 for a 95% confidence level; p is the estimated prevalence of shisha smoking within the population (50%, in order to produce the biggest sample size since there was no data on prevalence); e is the acceptable margin of error for the proportion being estimated (assumed to be 5%); and q = 1-p.

Therefore the sample size (n) was = 1.96^2^ × 0.5 × 0.5/ 0.05^2^ = 384.16. Adjustment for non-response of 10% was calculated by dividing the above sample size by 0.9 thus 384/0.9 = 427. Stratified sampling method was used [33], as students were stratified into schools or faculties and then into departments and finally, into program time of study (day, evening and weekend). Using a student’s list made available by each department on request, the number of students were chosen based on Probability Proportional to Size after inputting the list into Statistical Package for Social Sciences (SPSS) Version 20. Random selection was done using SPSS**.** Students were approached in lecture rooms shortly before or after a class. The questionnaires were distributed to the selected students, and the absent ones were replaced by running the SPSS again.

### Data collection

A self-administered questionnaire was adapted from a previous shisha study conducted in Kampala Uganda [16]. It was used to obtain information on prevalence of shisha use, knowledge about shisha, attitude towards use of shisha and factors associated with use of shisha. Information sought on prevalence was based on the participants response to the questions “Have you ever smoked shisha” and “Have you smoked shisha in the last month”. Ever smoked shisha and smoke shisha in the last month also referred to as current shisha smoking are recommended, standardized definitions of shisha prevalence [34]. Attitude was assessed using a Likert’s scale [35]. Responses were categorized as: strongly agree; agree; neither agree nor disagree; disagree and, strongly disagree (see Additional file 1).

The level of knowledge about shisha was assessed based on the ability of the participants to identify diseases associated with Shisha on a 6 point scale adopted from Viscusi Kenkel [36], and response to questions on whether shisha is a form of tobacco and whether it contains nicotine. Correct responses to questions were allocated a value of one giving a possible total of 8 points earmarked to the questions (see Additional file 2). Participants were then categorized as follows: good knowledge, with score of 6 or more; satisfactory knowledge, between 4 and 5 score; low knowledge, between 0 and 3 score.

### Data analysis

Collected data were entered into Epi-data software and imported into SPSS Version 20.0. The descriptive statistics included frequencies, and percentages. Cross tabulations using chi-square test was done to establish relationships between the independent variables and the dependent variable (shisha use). In this study, shisha use in the last month was considered as dependent or outcome variable. Significance level was considered at *p* value < 0.05. Multivariable analysis [37] was done with the use of binary logistic regression to determine variables that were independently significant by controlling the confounding variables. During the binary logistic regression, shisha use was defined or coded as *‘1’* and no shisha use was coded as *‘0’.*

### Ethical consideration

The study was authorized by the School of Postgraduate Studies and Research Mount Kenya University Rwanda. Before distributing the questionnaire, the researcher informed the students about the objectives of the study, and the confidentiality of their information. All participants had the right not to participate in the study.

## Results

### Socio-demographic and economic characteristics of the respondents

A total of 427 students were approached for interview of which 418 gave consent to participate (response rate of 97.9%)**.** Majority (63.1%) of the respondents were aged 20–24 years and the mean age of participants was 23.8 years. More females (57.7%) participated in the study. Most (84.7%) of the respondents were single and 84.4% of the respondents reside in the urban areas. The highest percentage (40.6%) was from the School of Business and Social sciences. Similarly the highest percentage were from the evening program (41.4%) followed by day program (37.5%). The result further revealed that majority (67.5%) of the respondents were employed and more than half (56.7%) belong to category 3 of the social class (Table 1). The categories were based on Rwanda’s socio-economic classification referred to as *Ubudehe* [38].

**Table 1:** Socio-demographic and economic characteristics of the respondents

Variable	Category	Frequency (*N* = 418)	Percentage (%)
Age in years	< 20	23	5.5
	20–24	264	63.1
	25–29	78	18.7
	30 & above	53	12.7
Gender	Male	177	42.3
	Female	241	57.7
Marital status	Divorced	13	3.1
	Single	354	84.7
	Married	51	12.2
Residence	Urban	353	84.4
	Rural	65	15.6
Faculty	School of Business &social sciences	170	40.6
	School of Pure & applied sciences	68	16.3
	School of Health sciences	165	39.5
	School of Education	15	3.6
Time of study	Day	157	37.5
	Evening	173	41.4
	Weekend	88	21.1
Educational level of father	Tertiary	247	59.1
	Secondary	139	33.3
	Primary	32	7.6
Educational level of mother	Tertiary	200	47.8
	Secondary	177	42.4
	Primary	41	9.8
Family’s social class	Category 4	38	9.1
	Category 3	237	56.7
	Category 2	122	29.2
	Category 1	21	5.0
Employment status	No	282	67.5
	Yes	136	32.5

### Prevalence of shisha use

Of the total of 418 respondents, 109 (26.1%) indicated that they had ever smoked shisha before while 87 (20.8%) said they had smoked shisha in the last month. “Smoked shisha in the last month” was used in data analysis and thus is the prevalence of focus in this study.

The main reasons for shisha smoking were curiosity 56 (51.4%), and peer influence 25 (23.0%) (Table 2).

**Table 2:** Shisha use among the respondents

Variables	Category	Frequency	Percentage (%)
		*N* = 418	
Ever smoked shisha	No	309	73.9
	Yes	109	26.1
Smoke shisha in the last month	No	331	79.2
	Yes	87	20.8
		*N* = 109	
Reason for smoking shisha	Curiosity	56	51.4
	Peer influence	25	23.0
	Stress/Anxiety	13	11.9
	To improve status	8	7.3
	Problem with study/friends	7	6.4

### Association of socio-demographic and economic characteristics with shisha use

Table 3 shows socio-demographic and economic characteristics stratified by shisha use. The table shows age (*p* = 0.002), sex (*p* = 0.007), marital status (*p* = 0.002), residence (*p* = 0.005), faculty (*p* < 0.001), mode of study (*p* < 0.001), and family’s social class (*p* = 0.018) were significantly associated with shisha smoking.

**Table 3:** Association of socio-demographic & economic characteristics with shisha use

Variable	Category	Shisha smoking		x^2^ test
		Yes (%)	No (%)	*P* value
Age in years	< 20	6(26.1)	17(73.9)	0.002
	20–24	58(22.0)	206(78.0)	
	25–29	22(28.2)	56(71.8)	
	30 & above	1(1.9)	52(98.1)	
Gender	Male	48(27.10)	129(72.9)	0.007
	Female	39(16.20)	202(83.8)	
Marital status	Divorced	6(46.2)	7(53.8)	0.002
	Single	78(22.0)	276(78.0)	
	Married	3(5.9)	48(94.1)	
Residence	Urban	82(23.2)	271(76.8)	0.005
	Rural	5(7.7)	60(92.3)	
Faculty	School of Business &social sciences	51(30.0)	119(70.0)	*0.000*
	School of Pure & applied sciences	20(29.4)	48(70.6)	
	School of Health sciences	14(8.5)	151(91.5)	
	School of Education	2(13.3)	13(86.7)	
Time of study	Day	23(14.6)	134(85.4)	*0.000*
	Evening	55(31.8)	118(68.2)	
	Weekend	9 (10.2)	79(89.8)	
Educational level of father	Tertiary	54(21.9)	193(78.1)	0.696
	Secondary	28(20.1)	111(79.9)	
	Primary	5(15.6)	27(84.4)	
Educational level of mother	Tertiary	45(22.5)	155 (77.5)	0.081
	Secondary	39(22.0)	138(78.0)	
	Primary	3(7.3)	38(92.7)	
Family’s social class	Category 4	15 (39.5)	23(60.5)	0.018
	Category 3	42(17.7)	195(82.3)	
	Category 2	27(22.1)	95(77.9)	
	Category 1	3(14.3)	18(85.7)	
Employment status	No	55(19.5)	227(80.5)	0.342
	Yes	32(23.5)	104(76.5)	

*p* value for Faculty has been italicized

*p* value for Time of study also italicized

### Knowledge and attitude according to shisha use

About three-quarters of the respondents, 313 (74.9%) indicated that they had heard about shisha.

About 40 % of the participants, 125 (39.9%), who had heard about shisha before demonstrated low level of knowledge while slightly lower proportion, 108 (34.5%) demonstrated satisfactory level of knowledge. Respondents with low knowledge were significantly more likely to use shisha than those with good knowledge (*p* < 0.001) (Table 4).

**Table 4:** Knowledge, attitude and shisha use

Variable	Category	Shisha smoking *N* = 418		Total	x^2^ test
		Yes (%)	No (%)	*N* = 418(%)	*p* value
Ever heard about shisha	No	0(0.0)	105(100)	105(25.1)	
	Yes	87(27.8)	226(72.2)	313(74.9)	
		*N* = 313		*N* = 313(%)	
Level of knowledge (N = 313)	Good	13(12.0)	95(88.0)	108(34.5)	*0.000*
	Satisfactory	22(27.5)	58(72.5)	80(25.6)	
	Low	52(41.6)	73(58.4)	125(39.9)	
		*N* = 418			
Attitude	Positive attitude	50(47.6)	55(52.4)	105(25.1)	*0.000*
	Neutral attitude	7(10.4)	60(89.6)	67(16.0)	
	Negative attitude	30(12.2)	216(87.8)	246(58.9)	

*p* value for Level of knowledge italicized

*p* value for Attitude italicized

Attitude was further classified into positive, neutral and negative attitude. More than half, 246 (58.9%) of the respondents had negative attitude towards shisha smoking. Respondents with positive attitude towards shisha were more significantly to use shisha than those with negative and neutral attitude (p < 0.001) (Table 4).

### Environmental, lifestyle, and behavioral factors stratified by shisha use

Most (70.3%) of the respondents were not drinking alcohol. Likewise, most (87.8%) of the respondents enjoy being in school. More than half (57.9%) of the respondents were participating in sport regularly. Majority (61.2%) were living with their parents and most of their parents (85.2%) were not smoking tobacco. Similarly, most siblings (58.6%) were not smoking any form of tobacco. Considerable percentage (40%) reported their friend(s) smoke shisha whereas 49.3% reported their friend(s) smoke conventional cigarette. It also highlights that most respondents (58.1%) were aware of shisha’s availability in cafes, bars and restaurants in Kigali (Table 5).

**Table 5:** Environmental, lifestyle, and behavioral factors stratified by shisha use

Variable	Category	Shisha smoking		Total	X^2^ test
		Yes (%)	No (%)	*N* = 418(%)	*P* value
Enjoy being in school	Yes	76(20.7)	291(79.3)	367(87.8)	0.711
	Not sure	7(25.9)	20(74.1)	27(6.5)	
	No	4(16.7)	20(83.3)	24(5.7)	
Regularly participate in sports	Yes	52(21.5)	190(78.5)	242(57.9)	0.691
	No	35(19.9)	141(80.1)	176(42.1)	
Drink Alcohol	Yes	35(44.3)	44(55.7)	79(18.9)	*0.000*
	Occasionally	13(28.9)	32(71.1)	45(10.8)	
	No	39(13.3)	255(86.7)	294(70.3)	
Lives with parent(s)	Yes	61(23.8)	195(76.2)	256(61.2)	0.056
	No	26(16.0)	136(84.0)	162(38.8)	
Sibling(s) smoke tobacco	Yes	21(29.6)	50(70.4)	71(17.0)	0.125
	No	48(19.6)	197(80.4)	245(58.6)	
	Don’t know	18(17.6)	84(82.4)	102(24.4)	
Parent(s) smoke tobacco	Yes	17(39.5)	26(60.5)	43(10.3)	0.006
	No	66(18.5)	290(81.5)	356(85.2)	
	Don’t know	4(21.1)	15(78.9)	19(4.5)	
Friend(s) smoke shisha	Yes	72 (43.1)	95(56.9)	167(40.0)	*0.000*
	No	15(6.0)	236(94.0)	251(60.0)	
Friend(s) smoke cigarette	Yes	65(31.6)	141(68.4)	206(49.3)	*0.000*
	No	22(10.4)	190(89.6)	212(50.7)	
Aware of shisha’s availability in cafes, bars in Kigali	Yes	81(33.3)	162(66.7)	243(58.1)	*0.000*
	No	6 (3.4)	169(96.6)	175(41.9)	

*p* value for Drink alcohol italicized

p value for Friend smoke shisha italicized

*p* value for Friend smoke cigarette italicized

*p* value for Aware of shisha's availability in cafes italicized

The bivariate analysis indicated that the following were significantly associated with shisha use: alcohol drinking (*p* < 0.001), respondents whose parents smoke tobacco (*p* = 0.006), respondents whose friends smoke shisha (*p* < 0.001), respondents whose friends smoke cigarette (*p* < 0.001) and respondents who were aware about shisha availability in cafes, restaurants and bars (*p* < 0.001) (Table 5).

### Factors independently associated with shisha use

Multiple logistic regression was applied to establish factors independently associated with shisha use (Table 6). All the factors associated with shisha use during bivariate analysis were considered together in the multivariable analysis. After running the multivariable analysis by specifying *backward conditional* method, the following five variables were independently associated with shisha smoking: age, always drink alcohol, occasionally drinks alcohol, friend(s) smoke shisha, aware of shisha’s availability in cafes, bars and restaurants in Kigali as well as attitude towards shisha.

**Table 6:** Factors independently associated with shisha use

Variable	Category	Shisha smoking		AOR	95% CI		*P* value
		Yes (%)	No (%)		Lower	Upper	
Age in years	< 20	6(26.1)	17(73.9)	15.24	1.152	201.761	0.039
	20–24	58(22.0)	206(78.0)	5.48	0.591	50.756	0.134
	25–29	22(28.2)	56(71.8)	8.18	0.841	79.544	0.070
	30 & above	1(1.9)	52(98.1)	Ref			
Drink Alcohol	Yes	35(44.3)	44(55.7)	3.03	1.475	6.216	0.003
	Occasionally	13(28.9)	32(71.1)	2.60	1.021	6.619	0.045
	No	39(13.3)	255(86.7)	Ref			
Friend(s) smoke Shisha	Yes	72(43.1)	95(56.9)	3.46	1.646	7.281	0.001
	No	15(6.0)	236(94.0)	Ref			
Aware of shisha’s availability in cafes, bars& rest. in Kigali	Yes	81(33.3)	162(66.7)	3.27	1.186	9.031	0.022
	No	6(3.4)	169(96.6)	Ref			
Attitude	Positive	50(47.6)	55(52.4)	3.32	1.697	6.478	*0.000*
	Neutral	7(10.4)	60(89.6)	0.85	0.291	2.479	0.766
	Negative	30(12.2)	216(87.8)	Ref			

*AOR* Adjusted Odds Ratio, *Ref* reference

*p* value for Attitude italicized

## Discussion

Our findings indicate that the prevalence of the students who reported to have ‘smoked shisha in the last month’ in a private university in Kigali city, Rwanda, was 20.8%. This is similar to prevalence rates for ‘smoked shisha in the last month’ that have been previously reported among youth in Estonia (21%), Czech Republic (22.1%), and in Latvia (22.7%) [39]. It also agrees with the findings of studies in Pakistan, where the prevalence of shisha use among students in a medical institution was reported as 21.5% [40], and in Lebanon, where the prevalence of current shisha smoking was 22.1% [41].

The prevalence of shisha smoking indicated in this study, however, is lower than those reported among youths in Kampala, where the prevalence of current shisha smoking was reported to be 36.4% [16], the reported prevalence of current shisha smokers among youth in Lebanon (36.9%) and in West Bank (32.7%) [39]. In Johannesburg, the prevalence of youth who reported that they had used shisha before or were using shisha at the time of the study was 60% [14], about three times the reported prevalence amongst the university students in Kigali City.

From the public health perspective, the findings of this study that the prevalence of shisha use is higher than the prevalence values that have been reported in studies among youth in other areas like in Southern California (10.7%) [42], United Arab Emirates (5.6%) [43] and Saudi Arabia (8.6%) [44], would suggest that an opportunity exists for the public health practitioners charged with the mandate of reducing the burden of shisha smoking among the youth to put in place intervention strategies that could lower the prevalence of shisha smoking amongst the youths.

It could also be important to have insights derive from studies on these communities why much lower prevalence values exist among the youths in those communities. It would also provide good practical lessons of public health significance to be learnt from those communities that the public health intervention strategists in the city of Kigali and other similar populations could possibly adapt and adopt.

The banning of shisha use in Rwanda has come after earlier bans in other East African countries like Uganda 2015 [45], and in Tanzania in 2016 [46]. Unfortunately, there has been widespread attention focused on the dangers of cigarette smoking and increasing efforts to discourage cigarette smoking, but comparatively less attention has been put to dangers of shisha use [47].

Our findings reveal that 60 % of the respondents had satisfactory and good knowledge about shisha and its negative effect. Although comparatively lower proportion, about 40 %, of the participants who had heard about shisha before demonstrated low level of knowledge about shisha, participants with low knowledge were shown to be more likely to use shisha than those with satisfactory knowledge and good knowledge.

This is supported by a study among adolescents in Saudi Arabia where participants with the knowledge score of < 4 were 56.3% among ever smokers and 38.2% among never smokers reflecting overall low knowledge about the health hazard of shisha smoking (*p* = 0.004) [48]. Among youths in Kampala, 67.7% of the participants had poor knowledge about shisha and its health effects [16].

The finding of this study is of importance to health promotion experts. Improving knowledge level on the dangers of shisha amongst the youth should be an important focus to those targeting the health of the youths in health promotion.

Consistent with previous reports [49–51], alcohol drinking (always drink alcohol and drink alcohol occasionally) was independently associated with shisha smoking. Also, having friends who smoke shisha was associated with shisha use. This supports the previous findings that having close friend(s) who smoked was identified as an important risk factor towards adolescents tobacco use among Iraqi students [52], and that more than half (59.2% females and 61.3% males participants) that were shisha smokers in a study in Kuwait said all or most of their friends smoked shisha [47].

In this study, it was found out that age below 20 years was independently associated with shisha use. This is in congruent with other studies where younger age was significantly associated with hookah smoking among U.S. students [53], among Southeast Michigan adults [54], and on a U.S. college campus [55]. Young people’s risk taking, experimentation, and vulnerability to peer pressure have been argued to be necessary parts of the individualization process, required for full identity achievement [56]. Public health intervention strategists should target these young age brackets in health education and promotion.

Participants who demonstrated positive attitude towards shisha were also associated with shisha smoking. This is consistent with studies in Kuwait where shisha smokers had more positive attitudes towards shisha smoking and were less likely to believe in its harmful effects [47], and among university students in Iraq where positive attitude towards smoking was reported as a significant predictor for current shisha smoking (*p* < 0.001) [51]. Public health intervention in the form of health promotion is needed to build the right attitude towards shisha use. It should be introduced early into the schools’ curriculum, and in collaboration with the media outlets, should emphasize on the detrimental effects and impending danger associated with shisha use.

The findings in this study indicated that curiosity, peer influence, stress/anxiety, to improve status, and problem with study/friends were reason for shisha smoking. Among university students in Pakistan, curiosity was found to be the most common reason (61.4%), followed by pleasure-seeking (46.9%), peer pressure (22.8%), boredom (17.8%) and stress (10.8%) [57]. In a University in Malaysia, shisha smoking was associated with problems among friends [58]. Peer pressure is a potent factor influencing smoking behavior. Understanding the dynamics of friendship patterns (both male and female) should be an important consideration in an effort to unravel the problems of youths’ health in this regard. This had been the focus of western social scientists for some time; however friendship patterns have not been studied among African youth. There is a need for studies on this topic, if effective programs to discourage smoking among young people are to be developed.

### Limitation

The findings of this study were based in Kigali city and the findings may not represent universities situated in non-urban districts. Another limitation was that the findings presented in this study were based on self-report and there was no way of ascertaining the reliability of the participants’ responses.

## Conclusion

Shisha is a popular form of tobacco smoking among these private university students in Kigali. This warrants increase shisha surveillance among this group and with the newly implemented ban on shisha use, advertisement and importation [15], measures should be put in place to effect strict implementation as well as impose fines on whoever flouts the regulations.

## Additional files

Additional file 1: Attitude of respondents towards shisha smoking. (DOCX 20 kb)

Additional file 2: Knowledge of shisha and its health effects. (DOCX 17 kb)

## Abbreviations

AIDS: Acquired Immunodeficiency Syndrome
AOR: Adjusted Odds Ratio
CI: Confidence Interval
HIV: Human Immunodeficiency Virus
MKU: Mount Kenya University
REF: Reference
SPSS: Statistical Package for the Social Sciences
U.S: United States
UK: United Kingdom
USA: United States of America
WHO: World Health Organization
