# Supplementary material for: Shisha use among students in a private university in Kigali city, Rwanda: prevalence and associated factors
Source: BMC Public Health. 2018 Jun 8;18:713. doi: 10.1186/s12889-018-5596-1 (PMC5994055; doi:10.1186/s12889-018-5596-1)
Supplement: Supplementary file 1 — Attitude of respondents towards shisha smoking. (DOCX 20 kb) [file 12889_2018_5596_MOESM1_ESM.docx]

**Additional file 1**

Table S1: Attitude towards shisha smoking (N=418)

| Variables | Category | Freq | Percent |
| --- | --- | --- | --- |
| Shisha is a safe habit | I strongly do not agree at all | 234 | 56.0 |
|  | I somewhat don’t agree | 35 | 8.4 |
|  | Neither agree nor disagree | 104 | 24.9 |
|  | I agree | 19 | 4.5 |
|  | I strongly agree | 26 | 6.2 |
| Shisha smoking is less addictive | I strongly do not agree at all | 122 | 29.2 |
|  | I somewhat don’t agree | 56 | 13.4 |
|  | Neither agree nor disagree | 151 | 36.1 |
|  | I agree | 51 | 12.2 |
|  | I strongly agree | 38 | 9.1 |
| Parent(s) approval of the habit | I strongly do not agree at all | 155 | 37.1 |
|  | I somewhat don’t agree | 54 | 12.9 |
|  | Neither agree nor disagree | 149 | 35.6 |
|  | I agree | 26 | 6.2 |
|  | I strongly agree | 34 | 8.1 |
| Shisha is less cancerous | I strongly do not agree at all | 103 | 24.6 |
|  | I somewhat don’t agree | 42 | 10.0 |
|  | Neither agree nor disagree | 177 | 42.3 |
|  | I agree | 61 | 14.6 |
|  | I strongly agree | 35 | 8.4 |
| More acceptable than cigarettes | I strongly do not agree at all | 100 | 23.9 |
|  | I somewhat don’t agree | 45 | 10.8 |
|  | Neither agree nor disagree | 154 | 36.8 |
|  | I agree | 68 | 16.3 |
|  | I strongly agree | 51 | 12.2 |
| Nicotine is lesser in shisha | I strongly do not agree at all | 98 | 23.4 |
|  | I somewhat don’t agree | 40 | 9.6 |
|  | Neither agree nor disagree | 180 | 43.1 |
|  | I agree | 52 | 12.4 |
|  | I strongly agree | 48 | 11.5 |
| Has less irritating smoke | I strongly do not agree at all | 107 | 25.6 |
|  | I strongly agree | 48 | 11.5 |
|  | Neither agree nor disagree | 159 | 38.0 |
|  | I agree | 67 | 16.0 |
|  | I strongly agree | 37 | 8.9 |
| Sign of high social status | I strongly do not agree at all | 132 | 31.6 |
|  | I somewhat don’t agree | 55 | 13.2 |
|  | Neither agree nor disagree | 156 | 37.3 |
|  | I agree | 45 | 10.8 |
|  | I strongly agree | 30 | 7.2 |
| Efficient filtration mechanism | I strongly do not agree at all | 96 | 23.0 |
|  | I somewhat don’t agree | 48 | 11.5 |
|  | Neither agree nor disagree | 204 | 48.8 |
|  | I agree | 49 | 11.7 |
|  | I strongly agree | 21 | 5.0 |
| Good stress-coping strategy | I strongly do not agree at all | 150 | 35.9 |
|  | I somewhat don’t agree | 65 | 15.6 |
|  | Neither agree nor disagree | 142 | 34.0 |
|  | I agree | 31 | 7.4 |
|  | I strongly agree | 30 | 7.2 |
| Shisha smoke has a sweet smell | I strongly do not agree at all | 113 | 27.0 |
|  | I somewhat don’t agree | 27 | 6.5 |
|  | Neither agree nor disagree | 148 | 35.4 |
|  | I agree | 50 | 12.0 |
|  | I strongly disagree | 80 | 19.1 |
| Fruit flavors detoxify smoke | I strongly do not agree at all | 98 | 23.4 |
|  | I somewhat don’t agree | 35 | 8.4 |
|  | Neither agree or disagree | 217 | 51.9 |
|  | I agree | 46 | 11.0 |
|  | I strongly agree | 22 | 5.3 |
| Classification of attitude | Positive attitude | 105 | 25.1 |
|  | Neutral attitude | 67 | 16.0 |
|  | Negative attitude | 246 | 58.9 |
| Freq= Frequency; Percent=Percentage (%) | | | |
