# Supplementary material for: Shisha use among students in a private university in Kigali city, Rwanda: prevalence and associated factors
Source: BMC Public Health. 2018 Jun 8;18:713. doi: 10.1186/s12889-018-5596-1 (PMC5994055; doi:10.1186/s12889-018-5596-1)
Supplement: Supplementary file 2 — Knowledge of shisha and its health effects. (DOCX 17 kb) [file 12889_2018_5596_MOESM2_ESM.docx]

**Additional file 2**

**Table S2: knowledge about Shisha and its health effects**

| Variable | Category | Freq(N=418) | Percent (%) |
| --- | --- | --- | --- |
| Ever heard about shisha | No | 105 | 25.1 |
|  | Yes | 313 | 74.9 |
|  |  | **N=313** |  |
| Shisha contains nicotine | No | 150 | 47.9 |
|  | Yes | 163 | 52.1 |
| Shisha is a form of tobacco | No | 81 | 25.9 |
|  | Yes | 232 | 74.1 |
| Hazard of Lung cancer | Yes | 163 | 52.1 |
|  | No | 44 | 14.0 |
|  | Don’t know | 106 | 33.9 |
| Hazard of Respiratory problems | Yes | 191 | 61.0 |
|  | No | 36 | 11.5 |
|  | Don’t know | 86 | 27.5 |
| Hazard of Cardiovascular problems | Yes | 115 | 36.7 |
|  | No | 49 | 15.7 |
|  | Don’t know | 149 | 47.6 |
| Hazard of Dental caries | Yes | 113 | 36.1 |
|  | No | 60 | 19.2 |
|  | Don’t know | 140 | 44.7 |
| Hazard of viral hepatitis | Yes | 53 | 16.9 |
|  | No | 68 | 21.7 |
|  | Don’t know | 192 | 61.4 |
| Hazard of Blindness | Yes | 26 | 8.3 |
|  | No | 83 | 26.5 |
|  | Don’t know | 204 | 65.2 |
| Level of knowledge | Good | 68 | 21.7 |
|  | Satisfactory | 86 | 27.5 |
|  | Low | 159 | 50.8 |
| Freq= Frequency; Percent=Percentage | | | |
